# Coexisting pituitary and non‐pituitary gigantism in the same family

**DOI:** 10.1111/cen.13852

**Published:** 2018-10-10

**Authors:** Pedro Marques, David Collier, Ariel Barkan, Márta Korbonits

**Affiliations:** ^1^ Centre for Endocrinology, William Harvey Research Institute, Barts and the London School of Medicine and Dentistry Queen Mary University of London London UK; ^2^ Department of Neurosurgery University of Michigan Ann Arbor Michigan

**Figure 1:**
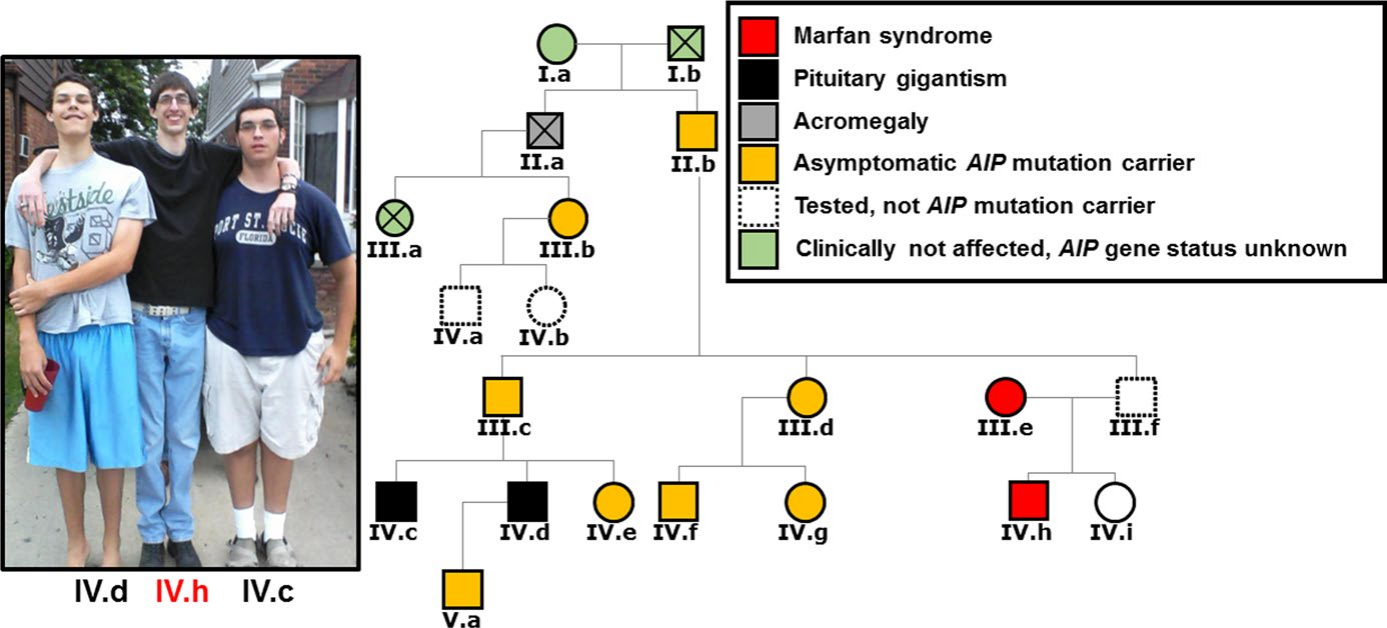
Pituitary gigantism due to *AIP* mutation‐positive pituitary adenomas in two brothers (IV.c, 196 cm; IV.d, 201 cm at the time of the photograph) and non‐pituitary gigantism due to Marfan syndrome in their first‐cousin (IV.h, 208 cm) [Colour figure can be viewed at http://www.wileyonlinelibrary.com]

Germline aryl hydrocarbon receptor‐interacting protein (*AIP*) mutations are present in 15%‐30% of familial isolated pituitary adenoma (FIPA) families, and are responsible for 30% of pituitary gigantism cases.^1^ However, pathological accelerated growth and/or tall stature can be unrelated to the growth hormone (GH) axis, and may occur in isolation or as part of a syndrome, such as in Klinefelter, Marfan or Sotos syndromes.^2^ Here, we report a five‐generation kindred with two brothers with pituitary gigantism due to *AIP* mutation‐positive GH‐secreting pituitary adenomas and their first‐cousin coincidently also having gigantism due to Marfan syndrome (Figure 1).

The proband, exhibited accelerated linear growth since he was 8 years‐old, and at the age of 10 measured 153 cm (height SDS + 2.1). He was diagnosed with pituitary gigantism due to a 2.5 cm GH‐secreting pituitary adenoma. Following transsphenoidal surgery, cabergoline and octreotide LAR were ineffective, and he was then successfully treated with pegvisomant^3^; his final height is 200 cm. His brother presented a few years later at the age of 16 with accelerated growth (height = 201 cm, SDS + 3.9), and he was operated on two occasions for a somatolactotropinoma. His final height is 209.5 cm.

Genetic testing identified a truncating heterozygous nonsense mutation in the *AIP* gene (c.910C>T; p.R304*) in both patients, as well as in eight unaffected family members, who are currently under surveillance. A deceased uncle had acromegaly based on photographs. In the same kindred there is a tall first‐cousin (height 208 cm) due to Marfan syndrome (Figure 1).

This is a unique family where two different diseases cause gigantism in close family members. The clinical diagnosis of the two brothers affected with GH‐related pituitary gigantism may have been hindered by the presence of extreme tall stature in this family due to Marfan syndrome. Clinical and biochemical exclusion of GH‐related pituitary gigantism is usually straightforward. However, the evaluation and management of patients with tall stature and/or acromegaloid features without GH/IGF‐1 axis abnormalities, i.e. pseudoacromegaly, may be challenging, particularly when the classical features of the underlying overgrowth syndrome are absent, there are coexistent acromegaloid features, biochemical discrepancies in the GH axis assessment, or family history of pituitary adenomas as illustrated here and elsewhere.^4^ Incidentally detected pituitary adenomas in these patients may further complicate the establishment of the correct underlying diagnosis.^4^ Overlapping features between GH excess and other conditions can present challenging issues for patients and their families as well as for their physicians.^4^, ^5^

Marfan syndrome is an autosomal dominant disorder caused by loss‐of‐function variants in the *FBN1* gene, mainly affecting the skeletal, ocular and cardiovascular systems. In Marfan syndrome, excessive linear growth of long bones starting at an early age results in tall stature, which justifies the assessment of GH axis. However, other typical manifestations such as joint laxity, long fingers, disproportionate long extremities for trunk size with increased arm span‐to‐height and lower‐to‐upper segment ratios, pectus carinatum or excavatum, as well as other features (myopia, ectopia lens, cardiovascular anomalies) should lead to an accurate diagnosis of Marfan syndrome.^2^

The diagnosis of many of the pseudoacromegaly conditions are usually established by experienced geneticists or dedicated paediatricians, but some of the conditions may be referred first to endocrinologists for investigation of possible GH excess or other hormonal disturbances affecting growth, such as precocious puberty, hypogonadism or thyrotoxicosis.^2^, ^5^ Therefore, endocrinologists should be aware of conditions masquerading as acromegaly/pituitary gigantism and thus be able to aid in establishing the underlying diagnosis.^4^, ^5^
